# One-Step Gas–Solid-Phase Diffusion-Induced Elemental Reaction for Bandgap-Tunable Cu_a_Ag_m1_Bi_m2_I_n_/CuI Thin Film Solar Cells

**DOI:** 10.1007/s40820-023-01033-5

**Published:** 2023-03-02

**Authors:** Erchuang Fan, Manying Liu, Kangni Yang, Siyu Jiang, Bingxin Li, Dandan Zhao, Yanru Guo, Yange Zhang, Peng Zhang, Chuantian Zuo, Liming Ding, Zhi Zheng

**Affiliations:** 1 Key Laboratory of Micro-Nano Materials for Energy Storage and Conversion of Henan Province, College of Chemical and Materials Engineering, Institute of Surface Micro and Nano Materials, Xuchang University, Xuchang, 461000 People’s Republic of China; 2 School of Materials Science and Engineering, Zhengzhou University, Zhengzhou, 450001 People’s Republic of China; 3 Center for Excellence in Nanoscience (CAS), Key Laboratory of Nanosystem and Hierarchical Fabrication (CAS), National Center for Nanoscience and Technology, Beijing, 100190 People’s Republic of China

**Keywords:** Cu_a_Ag_m1_Bi_m2_I_n_, Elemental reaction, Bandgap tuning, Solar cells, Gas–solid phase

## Abstract

Lead-free inorganic copper-silver-bismuth-halide materials have attracted more and more attention due to their environmental friendliness, high element abundance, and low cost. Here, we developed a strategy of one-step gas–solid-phase diffusion-induced reaction to fabricate a series of bandgap-tunable Cu_a_Ag_m1_Bi_m2_I_n_/CuI bilayer films due to the atomic diffusion effect for the first time. By designing and regulating the sputtered Cu/Ag/Bi metal film thickness, the bandgap of Cu_a_Ag_m1_Bi_m2_I_n_ could be reduced from 2.06 to 1.78 eV. Solar cells with the structure of FTO/TiO_2_/Cu_a_Ag_m1_Bi_m2_I_n_/CuI/carbon were constructed, yielding a champion power conversion efficiency of 2.76%, which is the highest reported for this class of materials owing to the bandgap reduction and the peculiar bilayer structure. The current work provides a practical path for developing the next generation of efficient, stable, and environmentally friendly photovoltaic materials.

**Supplementary Information:**

The online version contains supplementary material available at 10.1007/s40820-023-01033-5.

## Highlights

- The Cu_a_Ag_m1_Bi_m2_I_n_/CuI bilayer films are prepared simultaneously in situ by a one-step low-temperature gas-solid phase diffusion induced elemental reaction without spin coating.
- A new type of Cu_a_Ag_m1_Bi_m2_I_n_ photovoltaic material was originally designed to reduce the bandgap of this class of materials from 2.06 to 1.78 eV by breaking the restriction of double perovskite structure with a ratio of Ag:Bi = 1:1.
- The power conversion efficiency (PCE) of solar cell with a structure of FTO/TiO_2_/Cu_a_Ag_m1_Bi_m2_I_n_/CuI/carbon reached 2.76%, which is the highest PCE for Cu_a_Ag_m1_Bi_m2_I_n_ absorbers.

**Supplementary Information:**

The online version contains supplementary material available at 10.1007/s40820-023-01033-5.

## Graphical abstract

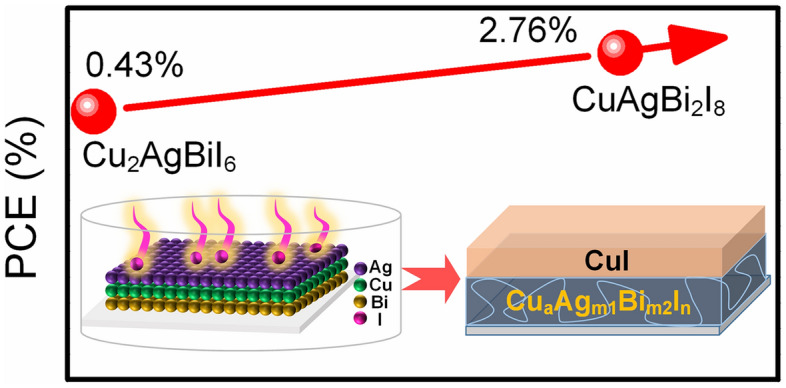

## Introduction

Considering comprehensive factors such as element abundance, cost, efficiency, environmental protection, and stability, the research and development of novel photovoltaic materials have never stopped [1–3]. The unique electronic structure of the Pb^2+^ plays a very critical role in the outstanding photovoltaic performances of perovskite materials [4–8], and it could be replaced by a suitable metal or multi-metal combination by a green chemical synthesis strategy, for the construction of environmentally friendly and high-quality inorganic photovoltaic thin films with ideal bandgaps, which have shown great application potential [9–14]. Particularly, isoelectronic Bi^3+^ possesses the same electron arrangement (6s^2^6p^0^) and similar ionic radius with Pb^2+^. From the point of view of the principle of charge balance (2Pb^2+^ → Ag^+^  + Bi^3+^), the lead-free Cs_2_AgBiBr_6_ double perovskite material based on the metal combination of Ag^+^ and Bi^3+^ has a good 3D structure, high absorption coefficient (≈ 10^5^ cm^−1^), long carrier lifetime, and high temperature/humidity stability, which provide the possibility and diversity for designing more ideal light-absorbing materials [10, 12, 15]. However, most of the reported Bi-based inorganic double perovskite materials show indirect bandgaps, and it is difficult to achieve the ideal bandgap values for single-junction solar cells [16].

In recent years, the design and performance optimization of perovskite-like Cu-Ag-Bi-based ternary halide photovoltaic materials with suitable bandgaps, such as CuBiI_4_ and Ag_1−3x_Bi_1+x_I_4,_ have attracted the attention of researchers [17–19]. However, due to the intrinsic influence of its own structure, the further improvement in both efficiency and stability is limited. In 2021, Rosseinsky's group [20] first reported a novel Cu_2_AgBiI_6_ multi-halide photovoltaic material inspired by the design of the double perovskite structure. The material has a direct bandgap of 2.06 eV, an ultra-high optical absorption coefficient of 1.0 × 10^5^ cm^−1^, a substantial charge-carrier mobility of 1.7 cm^2^V^−1^ s^−1^, and a long photoluminescence lifetime of 33 ns. However, the power conversion efficiency (PCE) is only 0.43% due to the large bandgap and poor film quality. Recently, it was found that the bandgap has little change by varying Cu^+^ ion content under the premise of keeping the Ag/Bi atomic ratio (1:1) [21]. Chang et. al. [22] introduced pyridine additives to improve the film quality and got a significant increase in PCE to 1.00% for Cu_2_AgBiI_6_ solar cell devices. And then, the crystallization kinetics of the film was controlled by adding 1.5 vol% HI, which improved the surface coverage and large crystallinity, resulting in an enhanced PCE of 1.3% [23]. As far as we know, the best efficiency of up to 2.39% is achieved by the hot-casting process [24]. Although such a new Cu-Ag-Bi-I-based compound has shown great potential as high-quality photovoltaic material, the current reported bandgap of ~ 2.0 eV still greatly deviates from the ideal bandgap [20–22, 25], which is still subject to structural design limitations with Ag/Bi ratio maintaining at 1:1. Another main problem lies in that the non-redox compound reaction among CuI, AgI, and BiI_3_ is prone to problems such as the random growth of secondary phases and poor film quality [20, 22–24].

While both free from the influence of lead and beyond the design concept of traditional double perovskite structure (Ag/Bi = 1:1), how to play the role of elemental chemistry to tune the optoelectronic properties of such Cu-Ag-Bi-I based compounds is a huge challenge for researchers. The electron transition from the electron-rich halogen to metal plays an important role in the spectral absorption of ionic crystals. The electron transition from I^−^ to Bi^3+^ is easier than that from I^−^ to Ag^+^ due to the higher oxidation number (III) of Bi^3+^. Thus, Bi-I coordination bond can absorb light with a longer wavelength, while the Ag-I coordination bond can absorb a shorter light. As a result, a smaller fraction of silver bismuth and a larger fraction of bismuth octahedra would be beneficial to reduce the bandgap. The fabrication of films based on metal elemental surface reaction is a kind of redox reaction with elemental material sputtered film as raw material, which is beneficial to tune the element ratio and improve the quality of film formation [17, 26–28].

In this work, the intelligently designed lead-free inorganic Cu_a_Ag_m1_Bi_m2_I_n_ absorber layer and the hole transport layer (CuI) were simultaneously fabricated by one-step gas–solid-phase diffusion-induced direct metal surface elemental reaction (DMSER) of copper-silver-bismuth-iodine at a low temperature. The bandgap of Cu_a_Ag_m1_Bi_m2_I_n_ could be reduced to 1.78 eV by modifying the sputtered Cu/Ag/Bi metal film thickness due to the destroy of the Ag/Bi ratio at 1:1. The CuAgBi_2_I_8_ solar cell with an FTO/TiO_2_/Cu_a_Ag_m1_Bi_m2_I_n_/CuI/carbon structure gained a champion efficiency of 2.76% for the single-junction solar cells. This approach of gas–solid-phase diffusion-induced elemental reaction is very simple and convenient, which presents a potential pathway to large-scale industrial production of Cu-Ag-Bi-I photovoltaic film materials.

## Experimental and Characterization

### Materials

The FTO substrates (1.5 × 1.5 cm^2^, 8 Ω) were purchased from Shanghai Zaofu New Materials Co., Ltd. Copper target (99%), bismuth target (99%), silver target (99%)) were purchased from Beijing Zhong Cheng Company. Acetone (C_3_H_6_O, 99.5%), isopropanol (C_3_H_8_O, 99.5%), anhydrous ethanol (C_2_H_6_O, 99.5%), and nitric acid (HNO_3_, 68%) were purchased from Sinopharm Chemical Reagent Co., Ltd. Carbon paste was obtained from Shanghai Mater Win New Materials Co., Ltd.

### Fabrication of Cu_a_Ag_m1_Bi_m2_I_n_/CuI Thin Film

First, the FTO was ultrasonic cleaned with detergent, deionized water, acetone, isopropanol, and anhydrous ethanol for 30 min, then blow-dried with N_2_, and irradiated with ultraviolet ozone for 30 min to remove organic matter on the surface. Second, 180 nm of Bi, 90 nm of Cu, and 60 nm of Ag thin films were deposited on the treated FTO substrates by a magnetron sputtering instrument under the Ar_2_ atmosphere. Third, 0.1 g of I_2_ particle and the sputtered Bi/Cu/Ag films were put in a sealed Teflon-lined stainless autoclave and heated at 100°C for 24 h. Finally, a gray-black CuAgBi_2_I_8_/CuI thin film was obtained on the FTO substrate. Cu_0.7_AgBi_2_I_7.7_ and Cu_0.6_AgBi_2_I_7.6_ thin films were fabricated by using the same procedure except for the thickness of Bi, Cu, and Ag. The Bi, Cu, and Ag thickness of Cu_0.7_AgBi_2_I_7.7_ films is 180, 60, and 60 nm, respectively. The Bi, Cu and Ag thickness of Cu_0.6_AgBi_2_I_7.6_ films is 120, 60, and 60 nm, respectively.

### Preparation of Cu_a_Ag_m1_Bi_m2_I_n_ Thin Film

The pure Cu_a_Ag_m1_Bi_m2_I_n_ film was obtained by etching CuI with 0.5 mol L^−1^ HNO_3_ for about 14 s.

### Fabrication of FTO/c-TiO_2_/m-TiO_2_/Cu_a_Ag_m1_Bi_m2_I_n_/CuI/C Solar Cell

The commercial FTO substrates with etched line were ultrasonically cleaned with detergent, deionized water, acetone, isopropanol, and anhydrous ethanol for 30 min in subsequence, then blow-dried with N_2,_ and irradiated with ultraviolet ozone for 30 min for removing organic on the substrate surface. 80 μL of c-TiO_2_ precursor was spin coated on the treated FTO substrates at the speed of 7000 rpm for 30 s, followed by annealing in air at 500°C for 2 h. The mesoscopic TiO_2_ (m-TiO_2_) layer was deposited by spin-coating the TiO_2_ colloid (30 s, 2000 rpm) and annealing it in air at 450°C for 30 min. After the fabricated TiO_2_ layer was treated by UV-ozone for 30 min, three metal monolayer films of Bi, Cu, and Ag metal thin film were sequentially deposited using a magnetron sputtering instrument under Ar atmosphere. And then, the sputtered metal films and iodine pellets were put into a sealed Teflon-lined stainless autoclave and heated at 100°C for 24 h to afford a structure of FTO/c-TiO_2_/m-TiO_2_/Cu_a_Ag_m1_Bi_m2_I_n_/CuI thin film. Finally, the commercial low-temperature conductive carbon paste was scraped onto the above prepared film with a blade and heated at 100°C for 10 min to prepare electrode with the active area of 0.04 cm^2^.

### Characterizations

The X-ray powder diffraction (XRD) patterns were obtained by using an X-ray diffractometer (Bruker, D8-Advanced) with Cu Kα radiation in a step size of 0.02°. Raman spectra were recorded by Raman spectrometer (Renishaw In Via). The X-ray photoelectron spectra (XPS) and ultraviolet photoemission spectra (UPS) were measured via an X-ray photoelectron spectrometer (ESCALAB Xi, Thermo Fisher Scientific). The top-view and cross-sectional morphologies, and element component were investigated through scanning electron microscopy (SEM) with an energy-dispersive X-ray (EDS) (Nova NanoSEM 50). The absorption curves were measured by a UV–vis-NIR spectrophotometer (Cary 5000 UV–Vis, Varian). The time-resolved photoluminescence (TRPL) was collected by a fluorescence spectrophotometer equipped with an excitation wavelength of 465 nm. The TSPV signals were collected by an oscilloscope (Tektronix TDS 3054C, 500 MHz) when the film was irradiated by a 355-nm pulsed laser. Current density–voltage characteristic (J-V) curves were measured in air under AM 1.5G sunlight generated by a solar simulator.

## Results and Discussion

### Fabrication and Characterization of Cu_a_Ag_m1_Bi_m2_I_n_ Films

**Fig. 1:**
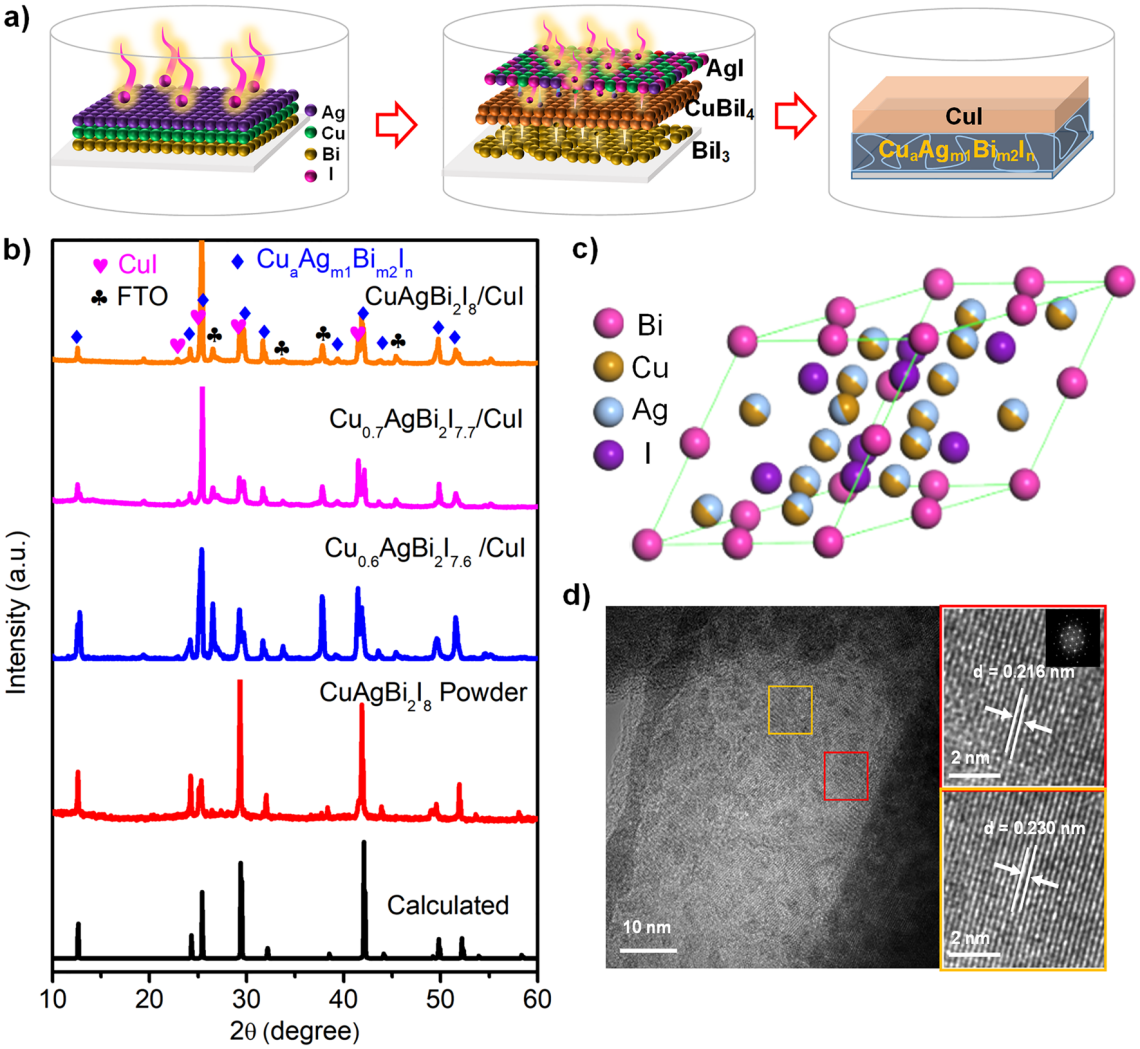
**a** Schematic of Cu_a_Ag_m1_Bi_m2_I_n_/CuI by the gas–solid-phase diffusion-induced direct metal surface elemental reaction (DMSER) of copper-silver-bismuth-iodine at a low temperature through tuning the Cu/Bi/Ag ratio. **b** XRD patterns spectra of Cu_a_Ag_m1_Bi_m2_I_n_/CuI, CuAgBi_2_I_8_ powder and calculated CuAgBi_2_I_8_ structure by MS software. **c** Crystal structure diagram of CuAgBi_2_I_8_ obtained by replacing Cu with Ag in the CuBiI_4_ structure. **d** HR-TEM image of CuAgBi_2_I_8_ and FFT pattern from red region and yellow region of CuAgBi_2_I_8_

A series of Cu_a_Ag_m1_Bi_m2_I_n_ quaternary compound films were prepared by the gas–solid-phase reaction method of copper-silver-bismuth-iodine at low temperature (Fig. 1a). In short, the definite thicknesses of Bi, Cu and Ag metal layers (the total metal thickness 240–330 nm) were successively sputtered on FTO glass in sequence (Figs. S1-S3), which reacted with excess iodine gas to afford Cu_0.6_AgBi_2_I_7.6_, Cu_0.7_AgBi_2_I_7.7_, and CuAgBi_2_I_8_ films at the bottom and CuI upper layer in a sealed Teflon-lined stainless autoclave. This pure phase CuAgBi_2_I_8_ powder was prepared by spin coating and annealing of iodide solution. The obtained samples were characterized by XRD. Figure 1b shows the XRD patterns of Cu_a_Ag_m1_Bi_m2_I_n_/CuI films, CuAgBi_2_I_8_ powder and the calculated structure by material studio (MS). These peaks at 22.8°, 25.2°, 29.7°, 41.5° should be assigned to the F-43 m cubic lattice of CuI (JCPDS No. 96–101-1240), indicating that a layer of CuI forms on the surface of CuAgBi_2_I_8_ film in the gas–solid-phase redox reaction, while the peaks at 12.6°, 24.1°, 25.5°, 29.1°, 38.6°, 42.8°, 49.7°, and 51.5° should be attributed to (111), (311), (222), (400), (333), (440), (622), and (444) planes of the relevant cubic (Fd $\overline{3 }$ m) phases of the CuAgBi_2_I_8_ quaternary compound. The structure of CuAgBi_2_I_8_ was obtained by replacing half of the Cu atoms with Ag atoms in CuBiI_4_ structure (ICSD No. 71533) (Fig. 1c), which is distinct with the alternating layer structure of Cu_2_AgBiI_6_ with both octahedral and tetrahedral sites filled with Cu^+^, Ag^+^, Bi^3+^, or I^−^ ions [20].

To clarify the structure difference between CuAgBi_2_I_8_ and Cu_2_AgBiI_6_, the upper CuI layer was etched by diluted HNO_3_ firstly. As shown in cross-sectional SEM images (Figs. S4-S6), the Cu_a_Ag_m1_Bi_m2_I_n_/CuI bilayer structure can transfer to single Cu_a_Ag_m1_Bi_m2_I_n_ layer after removing CuI by diluted HNO_3_ etching. Compared with the reported Cu_2_AgBiI_6_ structure [18, 29, 30], the typical peaks of CuAgBi_2_I_8_ at 2θ = 24.1°, 42.8°, and 51.5° shifted gradually to lower degrees (Fig. S7a), further confirming that the structure of CuAgBi_2_I_8_ is different from that of Cu_2_AgBiI_6_. The high-resolution transmission electron microscopy (HR-TEM) image of CuAgBi_2_I_8_ is shown in Fig. 1d. The clearly interplanar lattice spacings of 0.216 and 0.230 nm are indexed to crystal planes (440) and (333) of CuAgBi_2_I_8_, respectively. Therefore, the CuAgBi_2_I_8_ compound was successfully obtained. In the same preparation process, we regulate the ratio of Cu/Bi/Ag to prepared Cu_0.6_AgBi_2_I_7.6_, Cu_0.7_AgBi_2_I_7.7_ compound film. According to the XRD pattern of Cu_a_Ag_m1_Bi_m2_I_n_ films before and after etching (Fig. 1b and S7b), the peak positions of Cu_0.6_AgBi_2_I_7.6_, Cu_0.7_AgBi_2_I_7.7_, and CuAgBi_2_I_8_ have not changed except for the relative strength. Moreover, we further carried out a series of control experiments and compared the binary and ternary compounds with CuAgBi_2_I_8_ compound by XRD patterns (Fig. S8). The results show that there are no other phases in the absorption layer, and the absorption layer should be a single phase.

XPS was carried out to further investigate the composition of the Cu_a_Ag_m1_Bi_m2_I_n_ film after etching. The full XPS survey shows that all the expected elements Cu, Ag, Bi, and I were observed on the CuAgBi_2_I_8_ film after etching in Fig. S9. From the XPS spectra of Cu 2p of CuAgBi_2_I_8_ film and superficial CuI (Fig. S10), the two peaks with binding energies of 952.2 eV (2p_1/2_) and 932.2 eV (2P_3/2_) were observed obviously. The splitting of the spin orbit components is 20.1 eV, which is in accord with Cu^+^ [31, 32]. Furthermore, the Cu 2p peak positions of CuAgBi_2_I_8_ film are consistent with CuI, indicating that Cu^+^ is present in both CuI layer and CuAgBi_2_I_8_ layer. The Bi 4f_5/2_ and Bi 4f_7/2_ peaks at binding energies of 159.0 and 164.3 eV (Fig. S11) are associated with the iodide-bound bismuth (III) [33]. The Ag 3d spin orbitals (3d_5/2_ at 368.2 eV and 3d_3/2_ at 374.2 eV) with doublet separation of 6.0 eV can be assigned to Ag(I) (Fig. S12) [32]. Interestingly, the I core-level spectra show that the I 3d_3/2_ and 3d_5/2_ binding energies of CuAgBi_2_I_8_ shift 0.4 eV to the left in comparison to the CuI, revealing of the iodide-bound bismuth (Fig. S13). From the Raman spectra of Cu_0.6_AgBi_2_I_7.6_, Cu_0.7_AgBi_2_I_7.7_ and CuAgBi_2_I_8_ thin films (Fig. S14), the observed peak at 111.1 and 151.7 cm^−1^ should be assigned to Cu_a_Ag_m1_Bi_m2_I_n_ [20]. Interestingly, the peak position at 151.7 cm^−1^ never changes as the proportion of Cu/Ag/Bi elements, providing the further proof that the modified Cu_a_Ag_m1_Bi_m2_I_n_ quaternary compound should have the same crystal structure.

The evolution mechanism of CuAgBi_2_I_8_/CuI bilayer structure was studied by a series of controlled experiments. From the XRD patterns of CuAgBi_2_I_8_ compounds at different reaction time (Fig. S15), the typical peaks of CuBiI_4_, CuI, BiI_3_, and AgI could be found after one hour of reaction. When the reaction time was extended to 6 h, these peaks of CuAgBi_2_I_8_, CuBiI_4_, CuI, BiI_3_, and AgI coexisted on the XRD pattern. After 24 h of reaction, the pure Cu_a_Ag_m1_Bi_m2_I_n_/CuI bilayer structure was formed. The proposed reaction equation is as follows:

1 $${\text{2Ag }}\left( {\text{s}} \right){\mkern 1mu} + {\text{ I}}_{2} \;\left( {\text{g}} \right) \to {\text{2AgI }}\left( {\text{s}} \right)$$

2 $${\text{2Cu }}\left( {\text{s}} \right){\mkern 1mu} + {\text{ I}}_{2} \;\left( {\text{g}} \right) \to {\text{2CuI }}\left( {\text{s}} \right)$$

3 $${\text{Cu }}\left( {\text{s}} \right){\mkern 1mu} + {\text{ Bi }}\left( {\text{s}} \right){\mkern 1mu} + {\text{ 2I}}_{2} \;\left( {\text{g}} \right) \to {\text{CuBiI}}_{4} \;\left( {\text{s}} \right)$$

4 $${\text{2Bi }}\left( {\text{s}} \right){\mkern 1mu} + {\text{ 3I}}_{2} \;\left( {\text{g}} \right) \to {\text{2BiI}}_{3} \;\left( {\text{s}} \right)$$

5 $${\text{AgI }}\left( {\text{s}} \right){\mkern 1mu} + {\text{ BiI}}_{3} \;\left( {\text{s}} \right){\mkern 1mu} + {\text{ CuBiI}}_{4} \;\left( {\text{s}} \right) \to {\text{CuAgBi}}_{2} {\text{I}}_{8} \;\left( {\text{s}} \right)$$

The standard electrode potentials of Cu, Ag, and Bi are given by: E° (Ag^+^/Ag) = 0.80V, E° (Cu^+^/Cu) = 0.52V and E° (Bi^3+^/Bi) = 0.31V, respectively. Thus, the order of Bi, Ag, and Cu metal activation is Bi > Cu > Ag. Since Bi has the highest metal activation, we put it at the bottom to ensure that the reaction is complete. Due to the moderate activation of Cu, we tend to place the Cu under the Ag layer. As a result, a layer of CuI was formed on the CuAgBi_2_I_8_ indicating that there is a phenomenon of outward diffusion of Cu and inward diffusion of Ag driven by potential difference. The outer layer of Ag and the middle layer of Cu can firstly react with I_2_ gas to form AgI and CuI, respectively. Secondly, Bi and the extra Cu could further react with I_2_ gas to afford CuBiI_4_ during the atomic mutual diffusion. Thirdly, the BiI_3_ was formed by the reaction of the residual Bi and I_2_. Finally, the generated BiI_3_, AgI, and CuBiI_4_ were combined to form quaternary CuAgBi_2_I_8_ compound film at the bottom. Therefore, the CuAgBi_2_I_8_/CuI bilayer structure was formed mainly by the atomic mutual diffusion of Cu, Bi and Ag during the redox reaction.

**Fig. 2:**
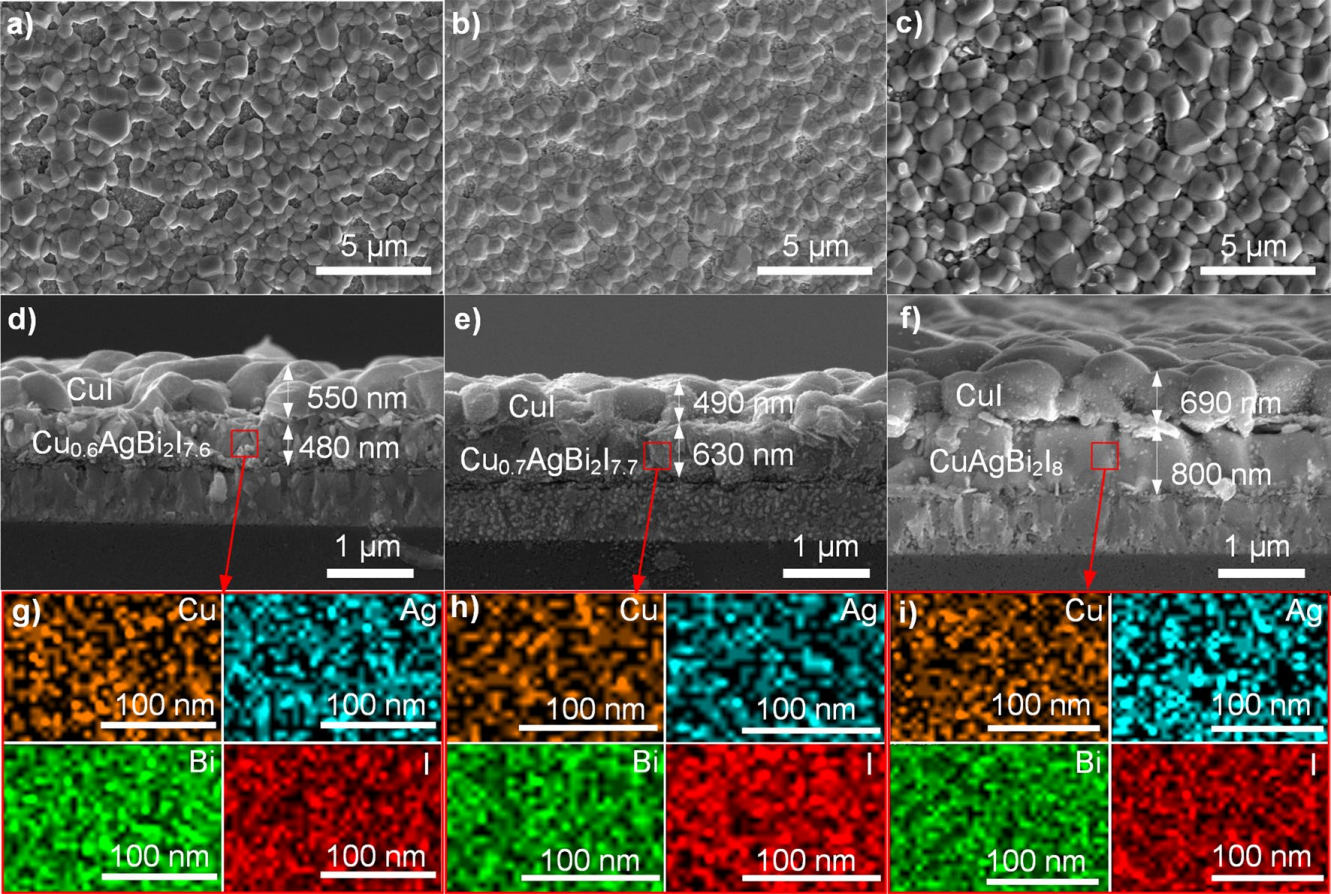
Top-view and cross-sectional SEM images. **a**, **d** for Cu_0.6_AgBi_2_I_7.6_, **b**, **e** for Cu_0.7_AgBi_2_I_7.7_, **c**, **f** for CuAgBi_2_I_8_ thin films on FTO substrates, respectively. SEM mapping images of Cu, Ag, Bi, and I elements for **g** Cu_0.6_AgBi_2_I_7.6_, **h** Cu_0.7_AgBi_2_I_7.7_, and **i** CuAgBi_2_I_8_

To determine the two-layered structure, scanning electron microscopy with energy-dispersive X-ray (SEM–EDX) was employed in characterization of morphology and composition analysis. As shown on the top-view SEM images (Fig. 2a, b, c), with the increase in the proportion of Cu in the compound, the upper grains gradually become larger and fuller, indicating the increase in Cu content can promote CuI growth. On the cross-sectional SEM images (Fig. 2d, e, f), the continuous and dense Cu_a_Ag_m1_Bi_m2_I_n _ and CuI bilayer structure can be obviously observed. The thickness of the top and the bottom layers was measured, about 550 and 480 nm for Cu_0.6_AgBi_2_I_7.6_/CuI, about 490 and 630 nm for Cu_0.7_AgBi_2_I_7.7_/CuI, and about 690 and 800 nm for CuAgBi_2_I_8_/CuI film, respectively. Take CuAgBi_2_I_8_ as an example, each layer of metal is about 60–180 nm, and the total sputtering thickness of Cu, Ag, and Bi is over 300 nm. On the one hand, to react with these metals, at least 600 nm of stacked iodine atoms is required since iodine atoms are approximately twice the total amount of Cu-Ag-Bi metals in terms of the atomic ratio of CuAgBi_2_I_8_ chemical formula. Therefore, the total thickness of Cu-Ag-Bi-I compounds may reach about 900 nm. On the other hand, the density difference between the metal and the compound must be taken into the account. Metals usually are denser than compounds. For example, Cu and CuI densities are about 9.0 and 5.6 g cm^−3^, respectively. And Bi and BiI_3_ densities are about 9.8 and 5.8 g cm^−3^, respectively. If the density of the metal is 1.6 times that of the compound, the total thicknesses of Cu-Ag-Bi-I compounds are roughly estimated to be 1440 nm, which is consistent with the observed thickness of Cu_a_Ag_m1_Bi_m2_I_n_/CuI films (1490 nm). Therefore, the thicknesses of Cu-Ag-Bi-I compounds and CuI are both over 400 nm.

The average composition of Cu_0.6_AgBi_2_I_7.6_, Cu_0.7_AgBi_2_I_7.7_, and CuAgBi_2_I_8_ was calculated statistically by SEM–EDX for no less than three regions (Tables S1-S3). The Ag/Bi ratio in all Cu_a_Ag_m1_Bi_m2_I_n_ is closed to 1:2. The SEM–EDX mapping images show a uniform distribution of Cu, Ag, Bi, and I elements (Fig. 2g, h, i). A small amount of Ag^+^ remained in the upper CuI layer. It is worth noting that the iodine content detected by EDX was lower than the expected value, which is attributed to the loss of iodine under the strong electron beam irradiation [23]. To further demonstrate the chemical composition, we scraped the powders of Cu_a_Ag_m1_Bi_m2_I_n_ (after etching) from FTO glass for inductively coupled plasma (ICP) and XPS tests. ICP results show that the atomic percentage of Cu, Ag, Bi, and I in CuAgBi_2_I_8_ is 9.70%, 8.41%, 17.4%, and 64.5% (Table S4), respectively. Thus, the composition of CuAgBi_2_I_8_ sample should be Cu_1.1_Ag_1.0_Bi_2.1_I_7.7_ by ICP measurement. At the same time, the compositions of Cu_0.7_AgBi_2_I_7.7_ and Cu_0.6_AgBi_2_I_7.6_ samples were measured to be Cu_0.69_Ag_1.0_Bi_1.8_I_8.1_ and Cu_0.58_Ag_1.0_Bi_2.0_I_8.1_, respectively, which are in line with the SEM–EDX results. The semi-quantitative analysis results of XPS also show that the atomic ratio of Ag to Bi is close to 1:2, which further proves the correctness of these compositions of Cu_a_Ag_m1_Bi_m2_I_n_ (Table S5). Therefore, the Cu_a_Ag_m1_Bi_m2_I_n_/CuI two-layered structure on the FTO was prepared successfully. The obtained CuI layer can be as a natural hole transport material of Cu_a_Ag_m1_Bi_m2_I_n_ absorption layer, which have excellent interfacial contact with the bottom layer [34].

### Optical Properties of Cu_a_Ag_m1_Bi_m2_I_n_ Films

**Fig. 3:**
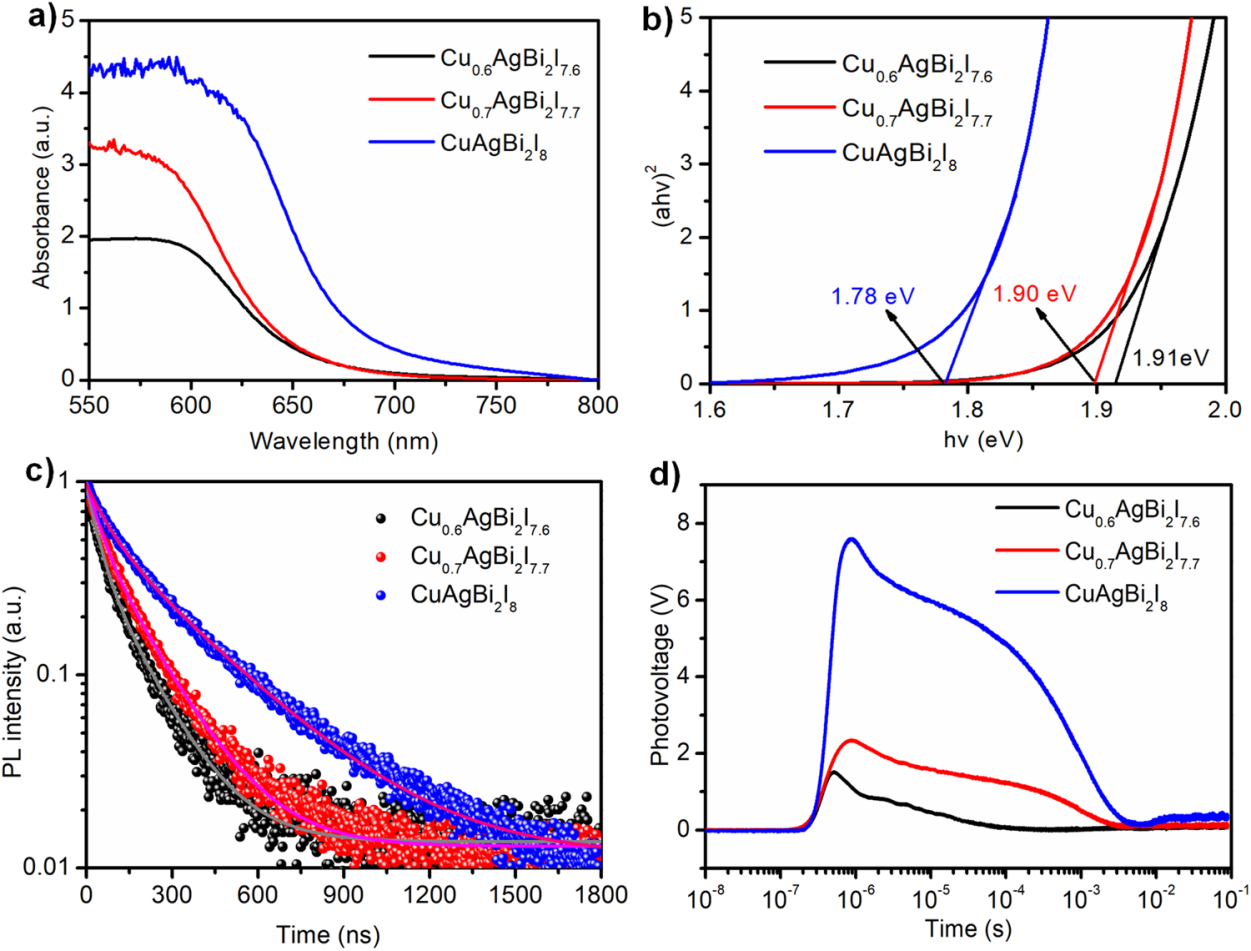
**a** UV–vis absorption spectra of Cu_a_Ag_m1_Bi_m2_I_n_ films after etching. **b** Corresponding (αhv)^2^ *vs* energy (hv) curves of Cu_a_Ag_m1_Bi_m2_I_n_ films after etching. **c** TRPL decay curves and **d** TSPV curves of Cu_a_Ag_m1_Bi_m2_I_n_ films after etching

The optical properties of Cu_a_Ag_m1_Bi_m2_I_n_ films after etching were characterized by UV–vis absorption spectra, TRPL decay curves, and transient surface photovoltage (TSPV) curves. As shown in Fig. 3a, with the increase in Cu content, the absorption edge shows red shift. According to the Tauc plot (Fig. 3b), the lowest value of bandgap is about 1.78 eV, 0.28 eV lower than the reported 2.06 eV [20]. Herz et al. [21] once tried to adjust the ratio of Cu by controlling the ratio of Ag/Bi to 1:1 and found that the bandgap only shifts by 0.05 eV from 2.05 eV for x = 0 (AgBiI_4_) down to 2.00 eV for x = 0.6 (Cu_6_AgBiI_10_). Thus, Cu content regulation cannot lead to such a large bandgap shift (0.28 eV). The modification of the ratio of Ag to Bi (atomic ratio Ag/Bi = 1:2) should play a key role on the bandgap regulation strategy. TRPL decay measurements show that the average carrier lifetime (τ_ave_) increased from 81.3 ns for Cu_0.6_AgBi_2_I_7.6_ film to 201 ns for CuAgBi_2_I_8_ film (Fig. 3c, Table S6). The PL lifetime of CuAgBi_2_I_8_ film is about 6 times that of the reported Cu_2_AgBiI_6_ film, indicating that the CuAgBi_2_I_8_ film has more advantages in charge separation. TSPV measurements of Cu_a_Ag_m1_Bi_m2_I_n_ films were carried out by using a 355-nm pulsed laser with a pulse width of 4 ns in according to the reported method [35]. A positive signal means that these Cu_a_Ag_m1_Bi_m2_I_n_ films have n-type semiconductor properties due to the accumulation of photoinduced electrons at the surface (Fig. 3d) [17, 35]. The photoinduced electron recombination time of CuAgBi_2_I_8_ film is about 5.48 × 10^−3^ s at the highest decay slope, which is higher than those of Cu_0.6_AgBi_2_I_7.6_ film (2.91 × 10^−3^ s) and Cu_0.7_AgBi_2_I_7.7_ (3.16 × 10^−4^ s). The photovoltage of CuAgBi_2_I_8_ film is the biggest among the Cu_a_Ag_m1_Bi_m2_I_n_ films, indicating of the highest photoinduced charge carrier concentration (Table S7). Charge carrier mobilities play a significant role in the performance of photovoltaic devices. We determined effective charge-carrier mobilities of CuAgBi_2_I_8_ by using Hall effect measurement. The value for the electron–hole total mobility is 2.80 cm^2^V^−1^ s^−1^ (Table S8), which is higher than that of double perovskite Cs_2_AgBiBr_6_ (0.8 cm^2^V^−1^ s^−1^) [36] and Cu_2_AgBiI_6_ (1.7 cm^2^V^−1^ s^−1^) [20, 25]. Charge carrier diffusion length is calculated by equation of $L_{D} = \sqrt {\tau \mu_{e} ekT/q}$, and the estimated value is 3.8 µm, indicating a preferable charge carrier diffusion length. Therefore, the CuAgBi_2_I_8_ film with a two-layered structure has the best dynamical behavior of photogenerated carriers, suggesting that it has a great application potential as an absorption layer in solar cell devices.

### Performance of Cu_a_Ag_m1_Bi_m2_I_n_ Solar Cells

**Fig. 4:**
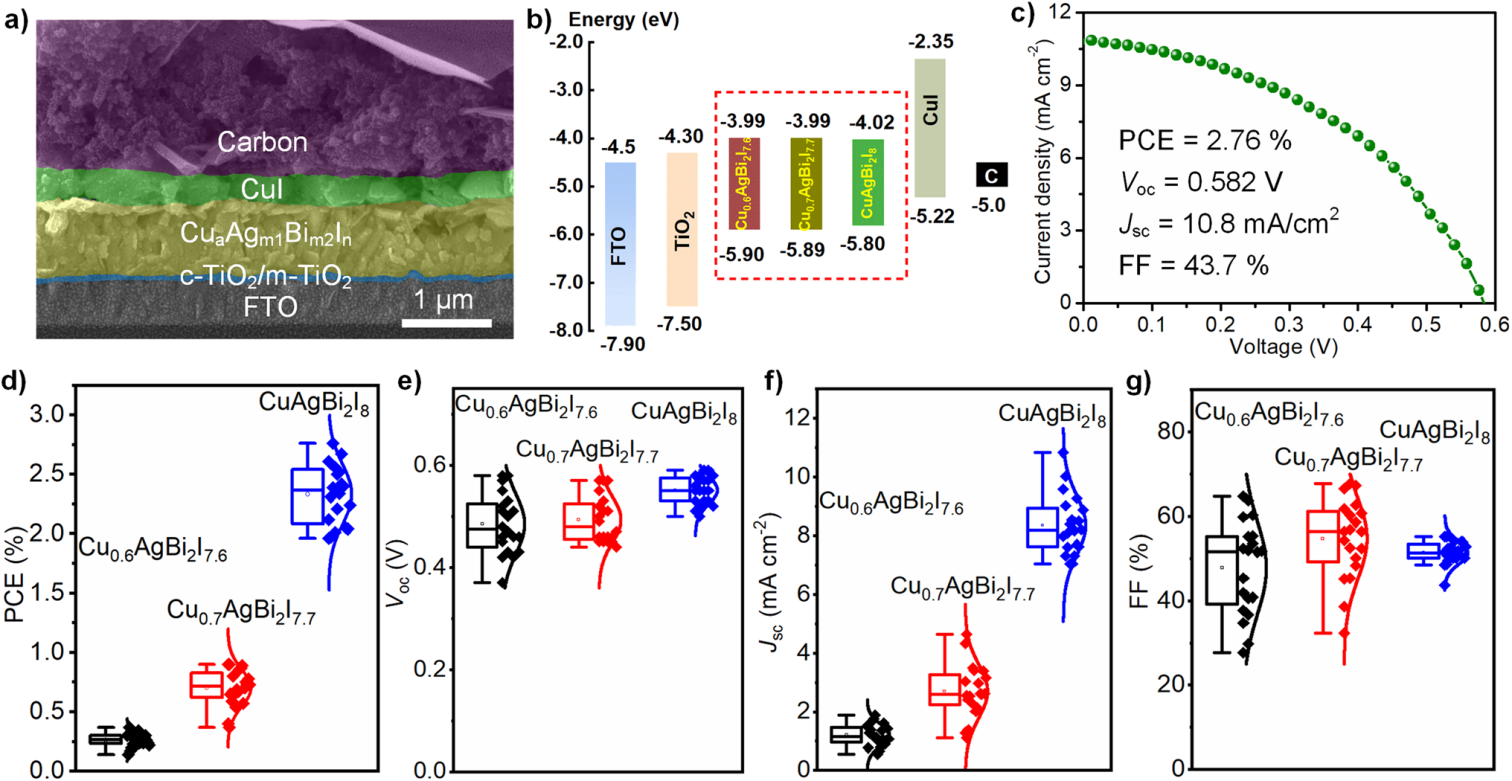
**a** Cross-sectional SEM image of the device with the architecture of FTO/c-TiO_2_/m-TiO_2_/Cu_a_Ag_m1_Bi_m2_I_n_/CuI/carbon. **b** Band alignment diagram of Cu_a_Ag_m1_Bi_m2_I_n_ film. **c** J-V plot of champion CuAgBi_2_I_8_ solar cell. The inset shows its J-V parameters PCE, V_oc_, J_sc_ and FF. **d** PCE, **e** V_oc_, **f** J_sc_ and **g** FF statistics of 20 devices under each condition. The box lines indicate the standard deviation, and the central represents the mean value

We assembled the CuAgBi_2_I_8_ film as an absorption layer into a planar heterojunction photovoltaic cell. Figure 4a shows the schematic cross-sectional view of the Cu_a_Ag_m1_Bi_m2_I_n_ solar cells. The n-i-p type cell has a structure of FTO/c-TiO_2_/m-TiO_2_/Cu_a_Ag_m1_Bi_m2_I_n_/CuI/carbon. A layer of activated carbon is deposited on Cu_a_Ag_m1_Bi_m2_I_n_ as a bifunctional film with a work function (− 5.0 eV) close to gold (− 5.1 eV), which can be effectively used for hole extraction and collection [37, 38]. In addition, carbon electrodes are very stable, processable, and inexpensive. The band structure of Cu_a_Ag_m1_Bi_m2_I_n_ was calculated through their UPS spectra (Figs. S16-S18) and UV–vis absorption spectra (Fig. 3a). As shown in Fig. 4b, band energy levels of the FTO, TiO_2_, Cu_a_Ag_m1_Bi_m2_I_n_, and carbon layers represent the smooth electron transport from the Cu_a_Ag_m1_Bi_m2_I_n_ to the TiO_2_ in the conduction band and hole extraction from the Cu_a_Ag_m1_Bi_m2_I_n_ to the CuI and carbon electrode in the valence band. Figure 4c shows the J-V curve of CuAgBi_2_I_8_/CuI based solar cell device. All the Cu_a_Ag_m1_Bi_m2_I_n_ solar cells with different ratio of Cu/Ag/Bi were also fabricated, as shown in Fig. S19. The corresponding photovoltaic parameters of the Cu_a_Ag_m1_Bi_m2_I_n_ solar cell devices with an active area of 0.04 cm^2^, including the short-circuit density (J_sc_), open-circuit voltage (V_oc_), fill factor (FF), and PCE, are presented in Fig. 4c and Table S9.

The PCE of CuAgBi_2_I_8_ solar cell is as high as 2.76%, which is highest value reported for this class of materials [20, 22, 24]. In detail, the CuAgBi_2_I_8_ solar cell has higher V_oc_ (0.582 V) and J_sc_ (up to 10.8 mA cm^−2^) than the reported Cu_2_AgBiI_6_ devices (Table S9). The J_sc_ data were almost twice as high as the reported (5.3 mA cm^−2^) [24]. All these are closely related to the bandgap regulation engineering, which enhances the harvest of light owing to the reduction in the bandgap. On the other hand, the CuI layer as a natural hole transport layer is beneficial to enhancing the hole extraction. As shown in Fig. S20, our CuAgBi_2_I_8_ devices show slight J–V hysteresis, which may be due to the imbalanced charge transportation at interfaces and non-radiative recombination [39]. To demonstrate the reproducibility and statistic properties, about 20 devices were fabricated and characterized as shown in Fig. 4d, e, f, g. The PCE statistics are distributed over a small range with an average value of 2.32%. This high photovoltaic performance of CuAgBi_2_I_8_ should be due to the growing of current density (J_sc_) caused by the regulation of Cu/Ag/Bi metal proportion (Fig. 4f). From the IPCE spectrum in Fig. S21, CuAgBi_2_I_8_ device has a wide spectral response in the wavelength range of 300–700 nm. An integrated current of CuAgBi_2_I_8_ is obtained from the IPCE spectra of the best cell. To furtherly evaluate the stability of Cu_a_Ag_m1_Bi_m2_I_n_ solar cell, the J-V curves of CuAgBi_2_I_8_ devices were recorded after 100 days in air (Fig. S22a). The unencapsulated Cu_a_Ag_m1_Bi_m2_I_n_ solar cell retains nearly 63% of its initial PCE after 100 days in air. The crystal structure of CuAgBi_2_I_8_ had not changed after 100 days in air by the determination of the XRD pattern (Fig. S22b).

## Conclusions

In summary, we fabricated a series of Cu_a_Ag_m1_Bi_m2_I_n_ films with self-generated CuI hole transport layer via the gas–solid-phase reaction of copper-silver-bismuth-iodine elements at low temperature. By tuning the sputtered Cu/Ag/Bi metal film thickness, the bandgap of the corresponding quaternary compounds could be reduced from 2.06 to 1.78 eV. Interestingly, both a continuous and dense Cu_a_Ag_m1_Bi_m2_I_n_ light absorption layer and a CuI hole transport layer could form simultaneously due to the strong atomic diffusion effect of Bi, Cu, and Ag during the redox reaction process. The CuAgBi_2_I_8_ solar cell with an FTO/TiO_2_/Cu_a_Ag_m1_Bi_m2_I_n_/CuI/carbon structure delivers an optimum PCE of 2.76% with an improved J_sc_ of 10.8 mA cm^−2^, which is the highest reported so far. This work will pave the way on the development of fabricating a new type environmentally friendly Cu_a_Ag_m1_Bi_m2_I_n_ inorganic photovoltaic materials. However, we have to admit that the current PCE is still too low in comparison to lead-based devices. At the present stage, we are not skilled in the dynamic control of this gas–solid reaction, with poor crystallinity, small grains, and many defects, leading to serious charge recombination. On the other hand, the device structure is only preliminary exploration, resulting that the interface charge transfer is not optimal. In addition, the bandgap has not reduced to an ideal range (1.4–1.6 eV). In the future, the photovoltaic properties of Cu_a_Ag_m1_Bi_m2_I_n_ thin film materials will be improved by introducing the coordination system and adjusting reaction activation energy to promote the generation of large crystal grains with little defects. By further adjusting the ratio of Cu, Ag, Bi, and I to tune the ratio of tetrahedral/octahedral lattice structure, the bandgap of Cu_a_Ag_m1_Bi_m2_I_n_ will be further adjusted to the ideal range.

### Supplementary Information

Below is the link to the electronic supplementary material.

Supplementary file1 (.docx 2.03 MB)
