# Supplementary material for: One-Step Gas–Solid-Phase Diffusion-Induced Elemental Reaction for Bandgap-Tunable CuaAgm1Bim2In/CuI Thin Film Solar Cells
Source: Nanomicro Lett. 2023 Mar 2;15:58. doi: 10.1007/s40820-023-01033-5 (PMC9981855; doi:10.1007/s40820-023-01033-5)
Supplement: Supplementary file 1 — Supplementary file1 (.docx 2.03 MB) [file 40820_2023_1033_MOESM1_ESM.docx]

**One**-**Step Gas**–**Solid**-**Phase Diffusion**-**Induced Elemental Reaction for Bandgap**-**Tunable Cu_a_Ag_m1_Bi_m2_I_n_/CuI Thin Film Solar Cells**

Erchuang Fan^1, 2^, Manying Liu^1^^,^ *, Kangni Yang^1^, Siyu Jiang^1, 2^, Bingxin Li^1^, Dandan Zhao^1^, Yanru Guo^1^, Yange Zhang^1^, Peng Zhang^2^, Chuantian Zuo^3^, Liming Ding^3,^ * and Zhi Zheng^1, 2,^ *

^1^ Key Laboratory of Micro-Nano Materials for Energy Storage and Conversion of Henan Province, Institute of Surface Micro and Nano Materials, College of Chemical and Materials Engineering, Xuchang University, Xuchang 461000, P. R. China

^2^ School of Materials Science and Engineering, Zhengzhou University, Zhengzhou 450001, P. R. China

^3^ Center for Excellence in Nanoscience (CAS), Key Laboratory of Nanosystem and Hierarchical Fabrication (CAS), National Center for Nanoscience and Technology, Beijing 100190, P. R. China

*Corresponding authors: E-mail: [manyingliu988@xcu.edu.cn](mailto:manyingliu988@xcu.edu.cn) (M. Y. Liu); [ding@nanoctr.cn](mailto:ding@nanoctr.cn) (L. M. Ding); [zzheng@xcu.edu.cn](mailto:zzheng@xcu.edu.cn) (Z. Zheng)

**Supplementary Figures and Tables**


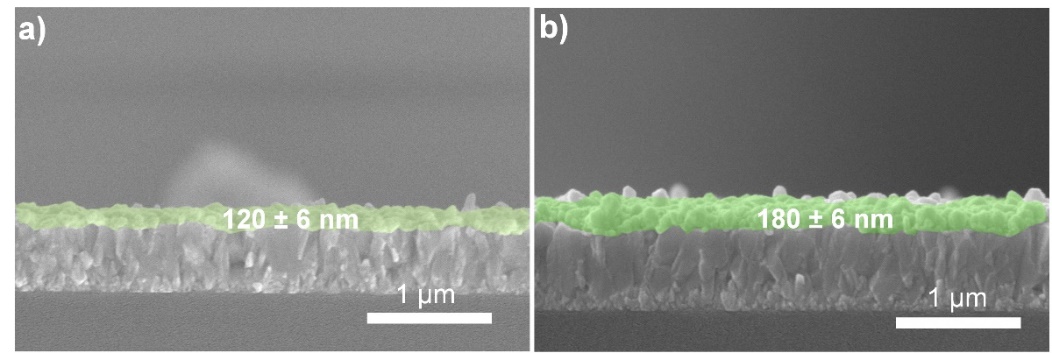


**Fig. S1** Cross-sectional SEM of sputtered Bi layers of (a)120 nm and (b) 180 nm


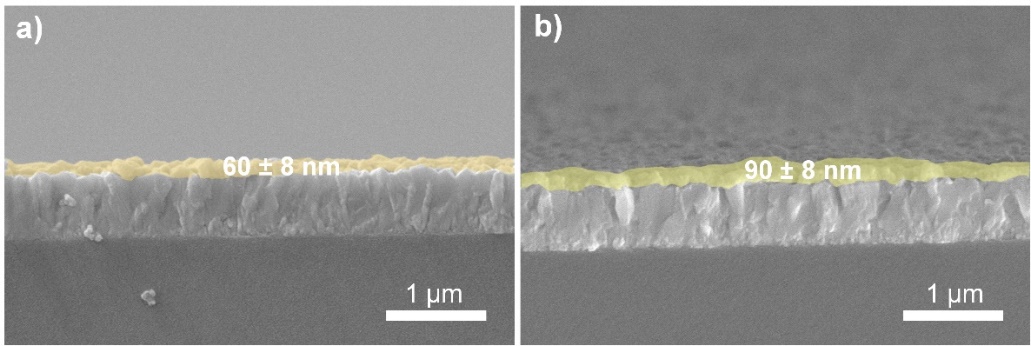


**Fig. S2** Cross-sectional SEM of sputtered Cu layers of (a) 60 nm and (b) 90 nm


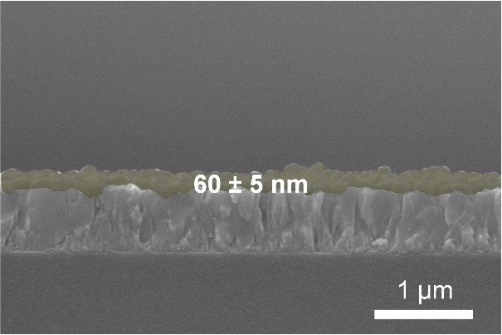


**Fig. S3** Cross-sectional SEM of sputtered Ag layers of 60 nm


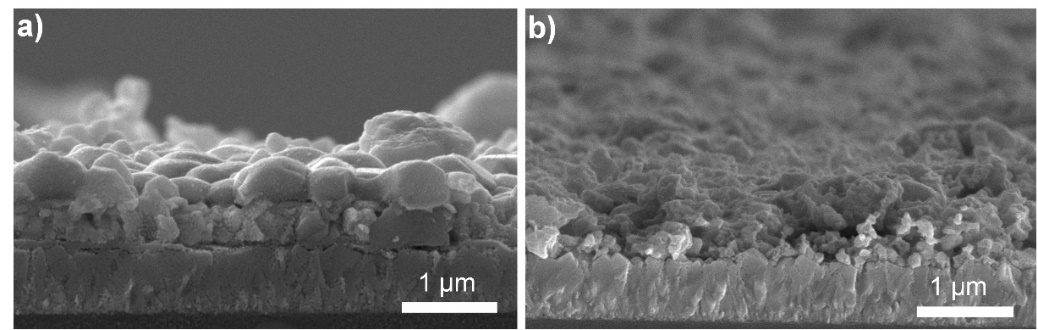


**Fig. S4** Cross-sectional SEM images of the Cu_0.6_AgBi_2_I_7.6_ film (a) before and (b) after etching by HNO_3_


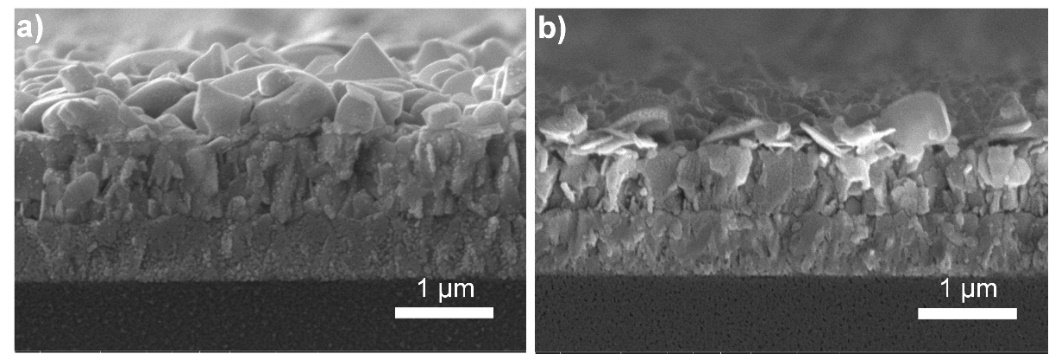


**Fig. S5** Cross-sectional SEM images of the Cu_0.7_AgBi_2_I_7.7_ film (a) before and (b) after etching by HNO_3_


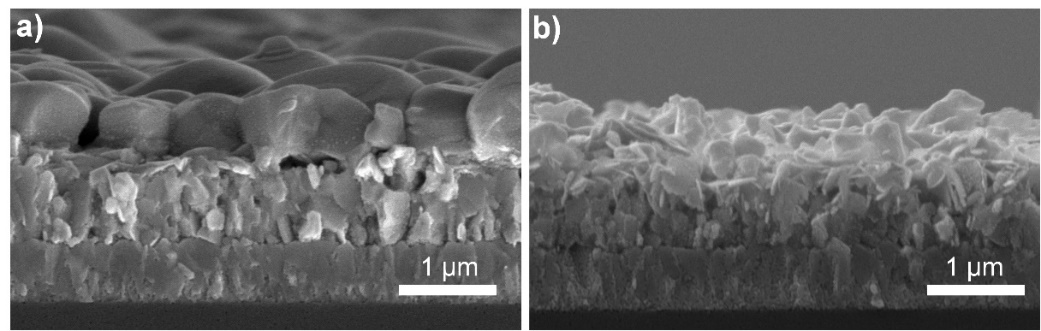


**Fig. S6** Cross-sectional SEM images of the CuAgBi_2_I_8_ film (a) before and (b) after etching by HNO_3_


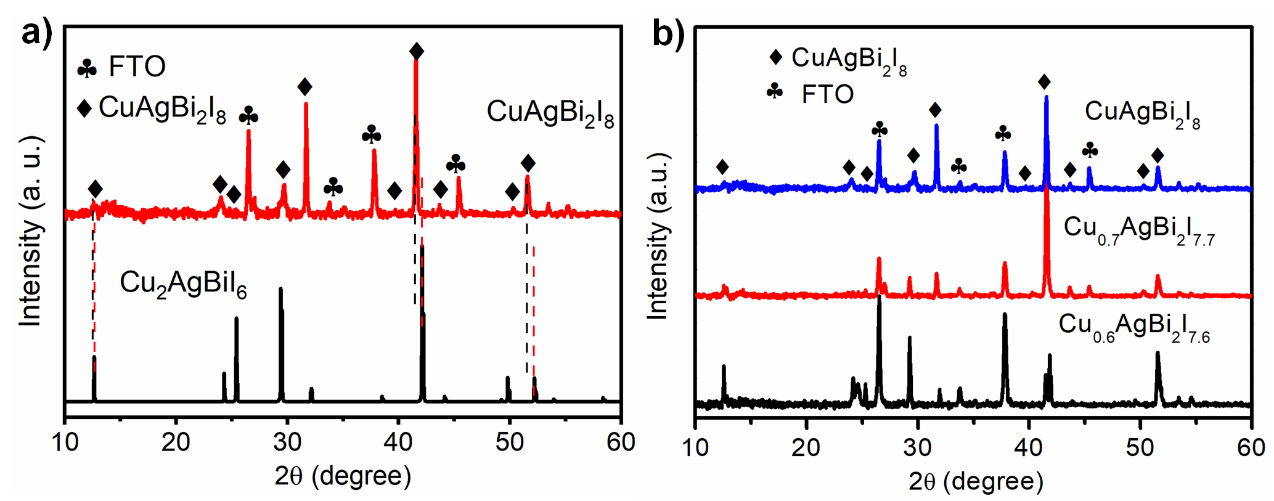


**Fig. S7** XRD patterns of (a) CuAgBi_2_I_8_ and Cu_2_AgBiI_6_, and (b) Cu_0.6_AgBi_2_I_7.6_, Cu_0.7_AgBi_2_I_7.7_, and CuAgBi_2_I_8_ after etching





**Fig. S8** XRD patterns of CuAgBi_2_I_8_, AgBiI_4_, CuBiI_4_, CuI, BiI_3_ and AgI





**Fig. S9** High resolution XPS spectra of CuAgBi_2_I_8_





**Fig. S10** High resolution XPS spectra of Cu 2p in CuAgBi_2_I_8_ and CuI





**Fig. S11** High resolution XPS spectra of Bi 4f in CuAgBi_2_I_8_





**Fig. S12** High resolution XPS spectra of Ag 3d in CuAgBi_2_I_8_





**Fig. S13** High resolution XPS spectra of I 3d in CuAgBi_2_I_8_ and CuI


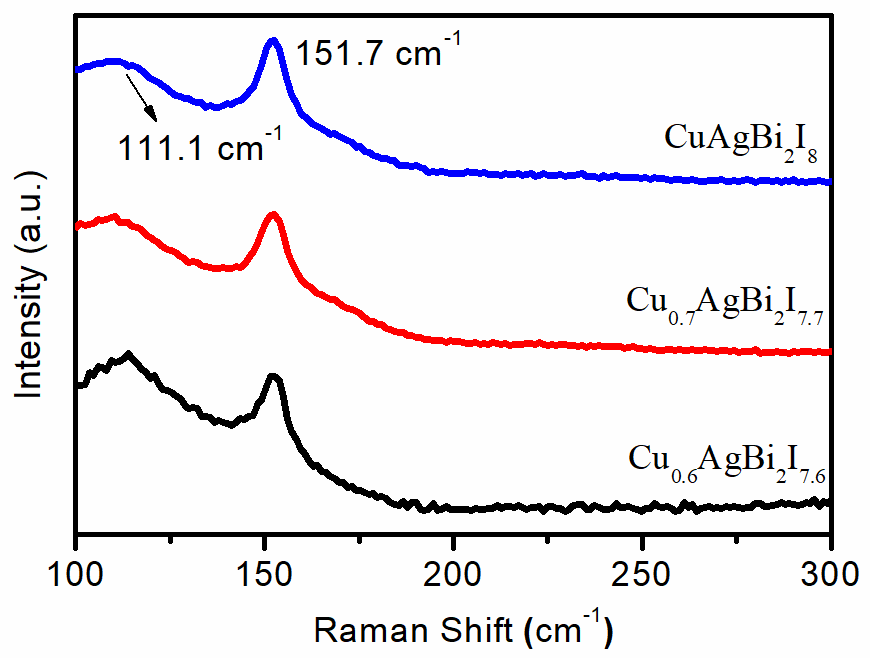


**Fig. S14** Raman spectra of Cu_0.6_AgBi_2_I_7.6_, Cu_0.7_AgBi_2_I_7.7_ and CuAgBi_2_I_8_ thin films





**Fig. S15** XRD patterns of CuAgBi_2_I_8_ compounds at different reaction time


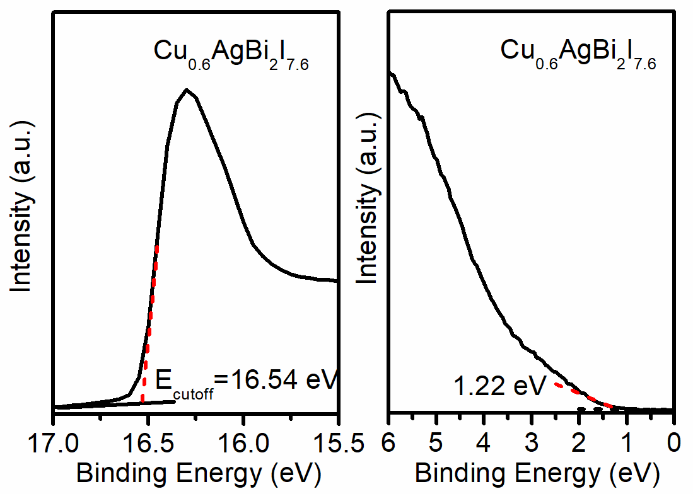


**Fig. S16** UPS spectra of Cu_0.6_AgBi_2_I_7.6_


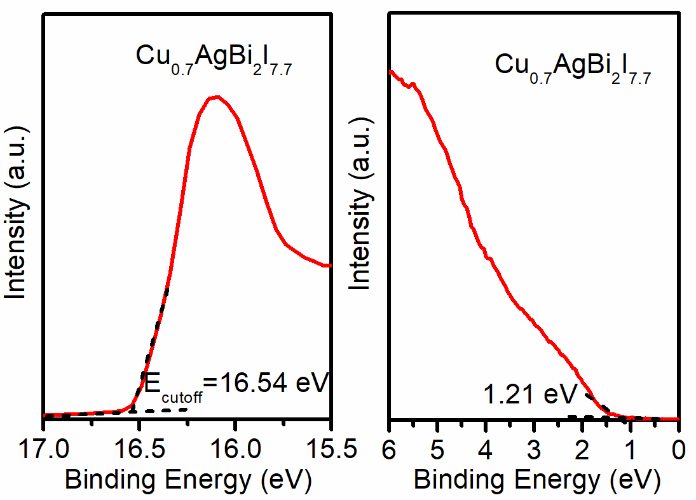


**Fig. S17** UPS spectra of Cu_0.7_AgBi_2_I_7.7_


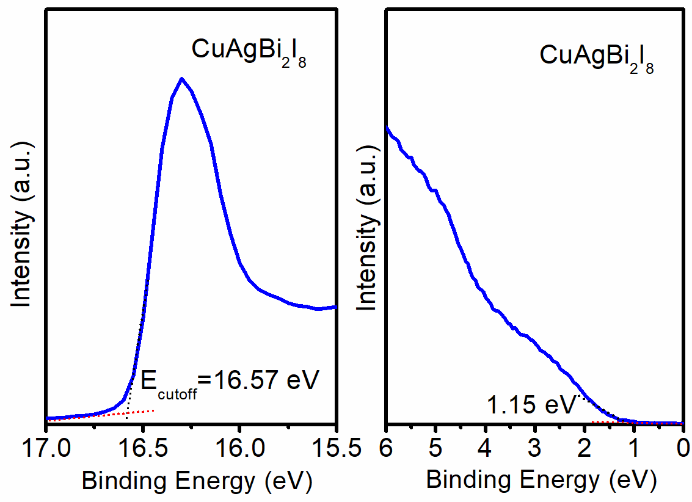


**Fig. S18** UPS spectra of CuAgBi_2_I_8_





**Fig. S19** J-V curve of Cu_0.6_AgBi_2.0_I_7.6_, Cu_0.7_AgBi_2_I_7.7_, and CuAgBi_2_I_8_ solar cells





**Fig. S20** J–V curves measured using CuAgBi_2_I_8_ devices under reverse and forward voltage scans at AM 1.5G illumination with a scan rate of 1·V·s^-1^





**Fig. S21** The IPCE spectra and integrated photocurrent of CuAgBi_2_I_8_ device


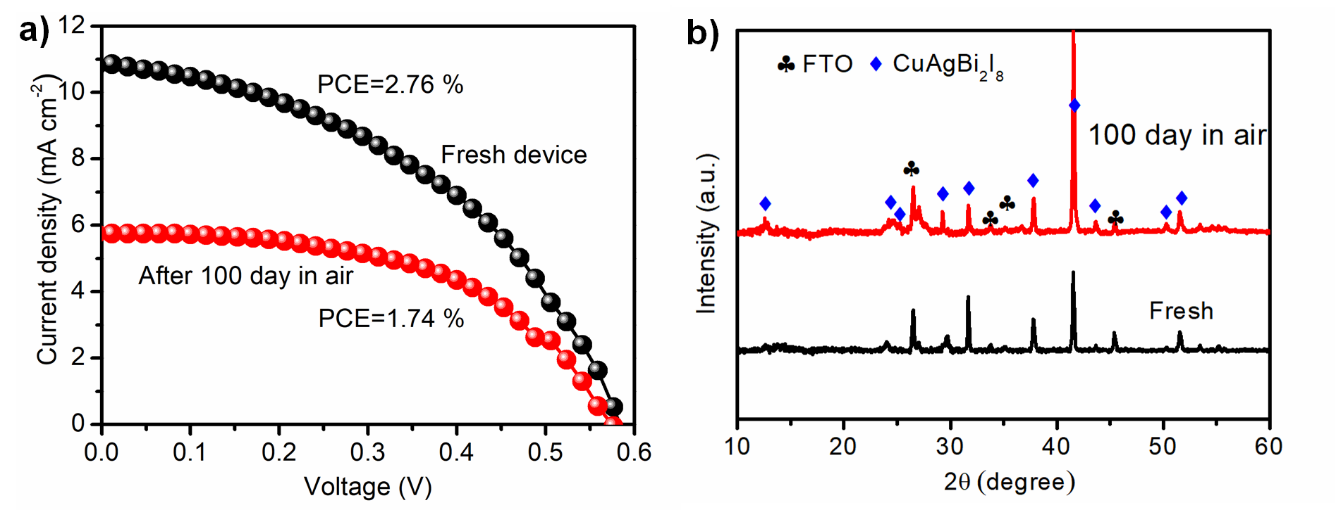


**Fig. S22** (a) The J-V curves of fresh CuAgBi_2_I_8_ device and CuAgBi_2_I_8_ devices after 100 days in air. (b) XRD patterns of fresh CuAgBi_2_I_8_ and CuAgBi_2_I_8_ film after 100 days in air

**Table S1** The Cross-sectional atomic ratios of Cu_0.6_AgBi_2_I_7.6_/CuI from EDS analysis

|  | Region | Cu (At%) | Ag (At%) | Bi (At%) | I (At%) |
| --- | --- | --- | --- | --- | --- |
| Top layer | 1 | 1.0 | 0.11 | 0.00 | 0.96 |
|  | 2 | 1.0 | 0.17 | 0.00 | 1.1 |
|  | 3 | 1.0 | 0.13 | 0.00 | 0.94 |
|  | Average | 1.0 | 0.14 ± 0.03 | 0.00 | 1.0 ± 0.1 |
| Bottom layer | 1 | 0.66 | 1.0 | 2.1 | 6.0 |
|  | 2 | 0.52 | 1.0 | 2.1 | 5.4 |
|  | 3 | 0.55 | 1.0 | 1.9 | 5.4 |
|  | Average | 0.60 ± 0.08 | 1.0 | 2.0 ± 0.1 | 5.6 ± 0.4 |

**Table S2** The Cross-sectional atomic ratios of Cu_0.7_Ag_1.00_Bi_2.00_I_7.7_/CuI from EDS analysis

|  | Region | Cu (At%) | Ag (At%) | Bi (At%) | I (At%) |
| --- | --- | --- | --- | --- | --- |
| Top layer | 1 | 1.0 | 0.07 | 0.00 | 1.0 |
|  | 2 | 1.0 | 0.07 | 0.00 | 1.0 |
|  | 3 | 1.0 | 0.09 | 0.00 | 1.1 |
|  | Average | 1.0 | 0.07 ± 0.02 | 0.00 | 1.0 ± 0.1 |
| Bottom layer | 1 | 0.75 | 1.0 | 1.9 | 5.9 |
|  | 2 | 0.75 | 1.0 | 2.0 | 5.7 |
|  | 3 | 0.68 | 1.0 | 2.1 | 6.1 |
|  | Average | 0.70 ± 0.08 | 1.0 | 2.0 ± 0.1 | 5.9 ± 0.2 |

**Table S3** The cross-sectional atomic ratios of CuAgBi_2_I_8_/CuI from EDS analysis

|  | Region | Cu (At%) | Ag (At%) | Bi (At%) | I (At%) |
| --- | --- | --- | --- | --- | --- |
| Top layer | 1 | 1.0 | 0.06 | 0.00 | 1.1 |
|  | 2 | 1.0 | 0.05 | 0.00 | 1.1 |
|  | 3 | 1.0 | 0.04 | 0.00 | 0.94 |
|  | Average | 1.0 | 0.05 ± 0.01 | 0.00 | 1.1 ± 0.1 |
| Bottom layer | 1 | 0.97 | 1.0 | 2.2 | 6.0 |
|  | 2 | 0.86 | 1.0 | 1.8 | 5.5 |
|  | 3 | 1.1 | 1.0 | 2.0 | 6.2 |
|  | Average | 1.0 ± 0.1 | 1.0 | 2.0 ± 0.2 | 5.9 ± 0.3 |

**Table S4** ICP analysis of Cu_a_Ag_m1_Bi_m2_I_n_

| Cu_a_Ag_m1_Bi_m2_I_n_ | Cu  (mol/L) | Cu (At%) | Ag (mol/L) | Ag (At%) | Bi (mol/L) | Bi  (At%) | I  (mol/L) | I (At%) |
| --- | --- | --- | --- | --- | --- | --- | --- | --- |
| Cu_0.6_AgBi_2_I_7.6_ | 0.70 × 10^-4^ | 5.00 | 1.2 × 10^-4^ | 8.57 | 2.4 × 10^-4^ | 17.1 | 0.97 × 10^-3^ | 69.4 |
| Cu_0.7_AgBi_2_I_7.7_ | 1.1 × 10^-4^ | 5.91 | 1.6 × 10^-4^ | 8.60 | 2.9 × 10^-4^ | 15.6 | 1.3 × 10^-3^ | 69.8 |
| CuAgBi_2_I_8_ | 1.5 × 10^-4^ | 9.70 | 1.3 × 10^-4^ | 8.41 | 2.7 × 10^-4^ | 17.4 | 1.0 × 10^-3^ | 64.5 |

**Table S5** The atomic ratios of Cu_a_Ag_m1_Bi_m2_I_n_ from XPS analysis

| Cu_a_Ag_m1_Bi_m2_I_n_ | Cu (At%) | Ag (At%) | Bi (At%) | I (At%) |
| --- | --- | --- | --- | --- |
| Cu_0.6_AgBi_2_I_7.6_ | 10.52 | 18.73 | 34.78 | 35.98 |
| Cu_0.7_AgBi_2_I_7.7_ | 8.000 | 11.83 | 24.13 | 56.04 |
| CuAgBi_2_I_8_ | 10.08 | 10.52 | 24.37 | 55.03 |

**Table S6** Fitting parameters of the TRPL spectra for Cu_a_Ag_m1_Bi_m2_I_n_ thin film

| Cu_a_Ag_m1_Bi_m2_I_n_ | A_1_ | τ_1_ (ns) | A_2_ | τ_2_ (ns) | τ_ave_ (ns) |
| --- | --- | --- | --- | --- | --- |
| Cu_0.6_AgBi_2_I_7.6_ | 0.430 | 143 | 0.500 | 39.6 | 81.3 |
| Cu_0.7_AgBi_2_I_7.7_ | 0.550 | 162 | 0.420 | 46.9 | 109 |
| CuAgBi_2_I_8_ | 0.580 | 299 | 0.370 | 73.2 | 201 |

**Table S7** The TSPV parameters of Cu_a_Ag_m1_Bi_m2_I_n_ thin film

| Cu_a_Ag_m1_Bi_m2_I_n_ | V_max_ (V) | T_t_ (s) | T_r_ (s) | T_t_/T_r_ |
| --- | --- | --- | --- | --- |
| Cu_0.6_AgBi_2_I_7.6_ | 1.51 | 5.23 × 10^-7^ | 3.16 × 10^-4^ | 1.66 × 10^-3^ |
| Cu_0.7_AgBi_2_I_7.7_ | 2.31 | 1.03 × 10^-6^ | 2.91 × 10^-3^ | 3.54 × 10^-4^ |
| CuAgBi_2_I_8_ | 7.57 | 9.59 × 10^-7^ | 5.48 × 10^-3^ | 1.75 ×10^-4^ |

**Table S8** The parameters of CuAgBi_2_I_8_ from Hall effect experiment

| Sample | Resistivity (ohm·cm) | Hall coefficient (cm^3^ C^-1^) | Carrier concentration  (cm^-3^) | Mobility  (cm^2^ v^-1^ s^-1^)) |
| --- | --- | --- | --- | --- |
| CuAgBi_2_I_8_ | 1.58 × 10^4^ | 4.44 × 10^4^ | 1.41 × 10^14^ | 2.80 |

**Table S9** Photovoltaic parameters for FTO/c-TiO_2_/m-TiO_2_/Cu_a_Ag_m1_Bi_m2_I_n_/CuI/C solar cells under AM 1.5 G irradiation

| Sample | V_oc_ (V) | J_sc_ (mA/cm^2^) | FF (%) | PCE (%) |
| --- | --- | --- | --- | --- |
| Cu_0.6_AgBi_2_I_7.6_ | 0.571 | 1.02 | 63.6 | 0.371 |
| Cu_0.7_AgBi_2_I_7.7_ | 0.510 | 2.62 | 67.4 | 0.902 |
| CuAgBi_2_I_8_ | 0.582 | 10.8 | 43.7 | 2.76 |
